# Complete chloroplast genome sequence of *Hopea chinensis* (Dipterocarpaceae), a rare and critically endangered species

**DOI:** 10.1080/23802359.2019.1681920

**Published:** 2019-11-18

**Authors:** Yancai Shi, Na Duan, Bingbing Liu

**Affiliations:** a Institute of Loess Plateau, Shanxi University, Taiyuan, Shanxi, China;; b Guangxi Institute of Botany, Guangxi Zhuang Autonomous Region and Chinese Academy of Sciences, Guilin, China

**Keywords:** *Hopea*, chloroplast genome, phylogenetic analysis

## Abstract

*Hopea chinensis* (Dipterocarpaceae) is a rare and critically endangered species. Here, we first report and characterize its complete chloroplast genome sequence based on Illumina paired-end sequencing data. The complete plastid genome was 152,054 bp, which contained inverted repeats (IR) of 21,702 bp separated by a large single-copy (LSC) and a small single-copy (SSC) of 20,462 bp and 88,188 bp, respectively. The cpDNA contains 129 genes, comprising 83 protein-coding genes, 38 tRNA genes, and 8 rRNA genes. The overall GC content of the plastome is 37.4%. The phylogenetic analysis of 18 selected chloroplast genomes demonstrated that *H. chinensis* was close to the species *Parashorea macrophylla*.

*Hopea chinensis* Hand. -Mazz., a precious tree which belongs to the family Dipterocarpaceae, is rare and critically endangered species in China. It is naturally distributed only in Guangxi (China) and Quang Ninh (Vietnam) (Trang and Triest 2016). However, because of the small number of populations and narrow habitats, *H. chinensis* is treated as rare and endangered species in China and has been registered on the China Species Red List (Fu 1991). It is thus urgent to take effective measures to conserve this critically endangered and precious species. Herein, we first report and characterize the complete plastome of *H. chinensis* based on Illumina paired-end sequencing data, which will contribute to the further studies on its genetic research and resource utilization. The annotated cp genome of *H. chinensis* has been deposited into GenBank with the accession number MN421794.

In this study, *H. chinensis* was sampled from the Guangxi Zhuang Autonomous Region of China, located at 110°10′14″ E, 25°43′35″ N. A voucher specimen (Y.-C. Shi et al. H028) was deposited in the Guangxi Key Laboratory of Plant Conservation and Restoration Ecology in Karst Terrain, Guangxi Institute of Botany, Guangxi Zhuang Autonomous Region and Chinese Academy of Sciences, Guilin, China. The experiment procedure is as reported in Zhang et al. (2019). Around 2 Gb clean data were used for the cp genome *de novo* assembly by the program NOVOPlasty (Dierckxsens et al. 2017) and direct-viewing in Geneious R11 (Biomatters Ltd., Auckland, New Zealand). Annotation was performed with the program Plann (Huang and Cronk 2015) and Sequin (http://www.ncbi.nlm.nih.gov/).

The chloroplast genome of *H. chinensis* is a typical quadripartite structure with a length of 152,054 bp, which contained inverted repeats (IR) of 21,702 bp separated by a large single-copy (LSC) and a small single copy (SSC) of 20,462 bp and 88,188 bp, respectively. The cpDNA contains 129 genes, comprising 83 protein-coding genes, 38 tRNA genes, and 8 rRNA genes. Among the annotated genes, 14 of them contain one intron (*clp*P, *ndh*A, *ndh*B, *rps*12, *rps*16, *rpoC*1, *pet*B, *pet*D, *rpl*2, *trn*A-UGC, *trn*I-GAU, *trn*K-UUU, *trn*L-UAA, and *trn*V-UAC), and one genes (*ycf*3) contain two introns. The overall GC content of the plastome is 37.4%.

To identify the phylogenetic position of *H. chinensis*, phylogenetic analysis was conducted. A neighbour-joining (NJ) tree with 1000 bootstrap replicates was inferred using MEGA version 7 (Kumar et al. 2016) from alignments created by the MAFFT (Katoh and Standley 2013) using plastid genomes of 17 species. It showed the position of *H. chinensis* was close to the species *Parashorea macrophylla* (Figure 1). Our findings can be further used for population genomic and phylogenomic studies of Dipterocarpaceae. It will also provide fundamental data for the conservation, utilisation, and management of this rare species.

**Figure 1.**
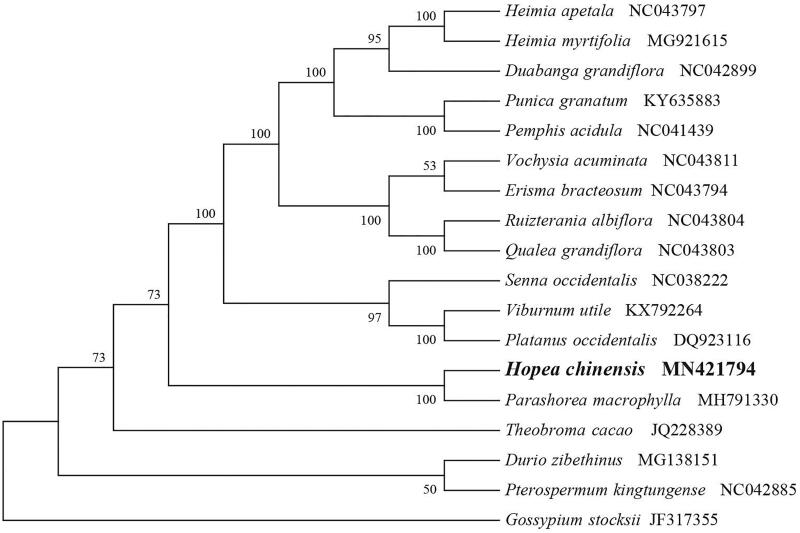
NJ phylogenetic tree of *H. chinensis* with 17 species was constructed by chloroplast plastome sequences. Numbers on the nodes are bootstrap values from 1000 replicates. *Gossypium stocksii* was selected as an outgroup.
